# Advances in Biosynthesis, Pharmacology, and Pharmacokinetics of Pinocembrin, a Promising Natural Small-Molecule Drug

**DOI:** 10.3390/molecules24122323

**Published:** 2019-06-24

**Authors:** Xiaoling Shen, Yeju Liu, Xiaoya Luo, Zhihong Yang

**Affiliations:** Institute of Medicinal Plant Development, Chinese Academy of Medical Sciences and Peking Union Medical College, Beijing 100193, China; 15189806892@163.com (X.S.); lyj18811358958@163.com (Y.L.); 18804897936@163.com (X.L.)

**Keywords:** pinocembrin, microbial biosynthesis, pharmacological activities, pharmacokinetic features, research progress

## Abstract

Pinocembrin is one of the most abundant flavonoids in propolis, and it may also be widely found in a variety of plants. In addition to natural extraction, pinocembrin can be obtained by biosynthesis. Biosynthesis efficiency can be improved by a metabolic engineering strategy and a two-phase pH fermentation strategy. Pinocembrin poses an interest for its remarkable pharmacological activities, such as neuroprotection, anti-oxidation, and anti-inflammation. Studies have shown that pinocembrin works excellently in treating ischemic stroke. Pinocembrin can reduce nerve damage in the ischemic area and reduce mitochondrial dysfunction and the degree of oxidative stress. Given its significant efficacy in cerebral ischemia, pinocembrin has been approved by China Food and Drug Administration (CFDA) as a new treatment drug for ischemic stroke and is currently in progress in phase II clinical trials. Research has shown that pinocembrin can be absorbed rapidly in the body and easily cross the blood–brain barrier. In addition, the absorption/elimination process of pinocembrin occurs rapidly and shows no serious accumulation in the body. Pinocembrin has also been found to play a role in Parkinson’s disease, Alzheimer’s disease, and specific solid tumors, but its mechanisms of action require in-depth studies. In this review, we summarized the latest 10 years of studies on the biosynthesis, pharmacological activities, and pharmacokinetics of pinocembrin, focusing on its effects on certain diseases, aiming to explore its targets, explaining possible mechanisms of action, and finding potential therapeutic applications.

## 1. Introduction

**Figure 1:**
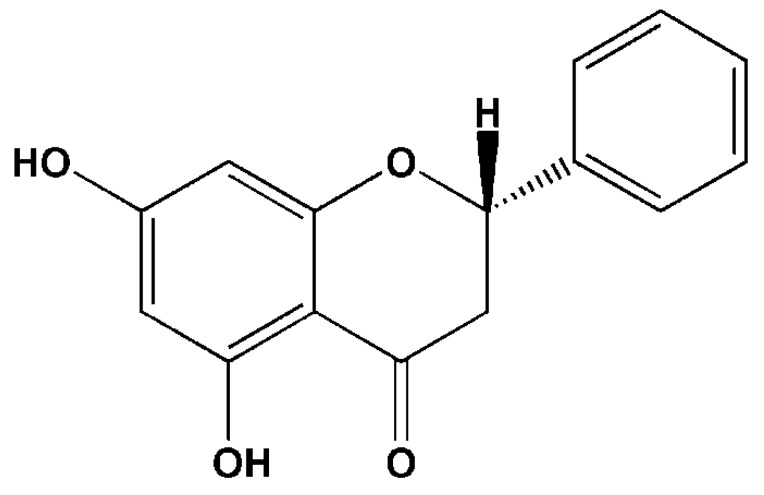
Chemical structure of (2S)-pinocembrin. Overall, this article reviews and expounds in detail the progress on pinocembrin biosynthesis, its pharmacological actions and partial mechanisms, and certain drug metabolism characteristics in vitro and in vivo, which will provide advantageous information for the comprehensive study of the pharmacokinetic (PK) features and pharmacological mechanisms of pinocembrin.

Pinocembrin is a pharmacologically active flavonoid that is mainly found in propolis, with a content reaching up to 606–701 mg/g in balsam which extracted from propolis with 70% ethanol [1]. Besides, it can be isolated from a variety of medicinal plants, such as *Peperomia* and *Piper* genera and *Asteraceae* families [2,3,4]. In addition to extraction from natural products, pinocembrin can be synthesized by biological and chemical methods. Biosynthesis plays an important role in the synthesis of pinocembrin owing to its high yield and low production cost. In terms of pharmacological effects, pinocembrin can exhibit anti-inflammatory [5], anti-oxidant [6], antibacterial [7] and neuroprotective activities [8]. Research has shown that pinocembrin exhibits a positive effect on the treatment of ischemic stroke. Pinocembrin can reduce the area of cerebral infarction in rats with cerebral ischemia and reduce the degree of cerebral edema and apoptosis of nerve cells. In addition, pinocembrin can protect the integrity of the blood–brain barrier (BBB), thereby reducing mortality and improving neurobehavioral scores in rats [9]. Due to its significant pharmacological activity, pinocembrin has been approved by China Food and Drug Administration (CFDA) as a new drug for treatment of ischemic stroke, now in Phase II clinical trials. Recent studies have also revealed that pinocembrin possesses an anti-tumor effect, such as against melanoma [10]. It also shows anti-fibrosis [11] effect. An excellent drug not only possesses good pharmacological activities but also good pharmacokinetic (PK) parameters. Pinocembrin features a small molecular weight (Figure 1) and good liposolubility, allowing it to easily pass through the BBB. The transport mode of pinocembrin may primarily transpire by passive transport. This property suggests that this candidate drug can be used for treatment of brain diseases [12]. Both clinical and preclinical experiments have shown that pinocembrin can be absorbed quickly and distributed widely without notable accumulation of residues, indicating that this compound possesses good PK profiles. Thus, pinocembrin is a potential natural small-molecule drug with good prospects for further development.

## 2. Microbial Biosynthesis

### 2.1. Synthesis of Pinocembrin from Glucose

**Figure 2:**
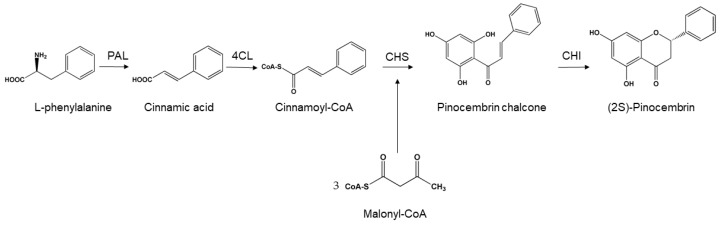
Classic biosynthetic pathway of pinocembrin [15]. L-Phenylalanine as a precursor compound produces cinnamic acid under the action of phenylalanine ammonia lyase, which generates cinnamoyl-CoA under the action of CoA ligase, and adds malonyl-CoA to the reaction system to form pinocembrin chalcone under the action of chalcone synthase. Finally, pinocembrin is generated under the action of chalcone isomerase.

Pinocembrin can be extracted from natural products but at a high production cost and insufficient yield. Microbial biosynthesis features the advantages of low cost and large output, which compensate for the lack of natural sources of pinocembrin. *Escherichia coli* is widely used in producing pinocembrin. In recent years, researchers have been working on how to produce pinocembrin efficiently. Biosynthesis of pinocembrin often require supplementation of expensive phenylpropanoic precursors (Figure 2), presenting a major problem in the past studies. To solve this issue, genetic engineering is used to construct engineering bacteria in order to synthesize pinocembrin from glucose. In order to produce the flavonoid precursor (2*S*)-pinocembrin directly from glucose, 3-deoxy-D-arabinoheptulosonate-7-phosphate synthase, chorismate mutase/prephenate dehydratase, phenylalanine ammonia lyase (PAL), 4-coumarate:CoA ligase (4CL), chalcone synthase (CHS), chalcone isomerase (CHI), malonate synthetase, and malonate carrier protein have been assembled in four vectors. Synthesizing pinocembrin from glucose can be realized with the adjustment of other corresponding conditions [13,14].

### 2.2. Production Optimization Measures of Pinocembrin

Several key factors may affect the production efficiency of pinocembrin. First, *E. coli* metabolites affect the synthesis of pinocembrin. The production efficiency of pinocembrin is limited by the content of malonyl-CoA. However, in *E. coli*, both the biosynthesis of pinocembrin and self-fatty acid biosynthesis of the bacterium consume malonyl-CoA. Thus, limiting *E. coli* self-fatty acid synthesis can improve the synthetic efficiency of pinocembrin. Research has shown that overexpressing enzymes β-ketoacyl-acyl carrier protein synthase III (FabH), FabF, or both enzymes in *E. coli* BL21 (DE3) decreased fatty acid synthesis and increased cellular malonyl-CoA levels, thereby up-regulating the production of pinocembrin [16]. In addition, the accumulation of cinnamic acid adversely affects the production of pinocembrin. Screening gene sources and optimizing gene expression are employed to regulate the synthetic pathway of cinnamic acid. Then, site-directed mutagenesis of chalcone synthase and cofactor engineering are used to optimize the downstream pathway of cinnamic acid consumption. These strategies reduce the accumulation of cinnamic acid and increase the yield of pinocembrin [17].

Adenosine triphosphate (ATP) is the source of energy for various activities in living organisms. Therefore, ATP concentration in *E. coli* must be controlled. To screen several ATP-related candidate genes, a clustered regularly interspaced short palindromic repeats (CRISPR) interference system has been established. MetK and proB have been found to show potential in improving ATP level and increasing the production of pinocembrin [18]. Different culture pH values during microbial fermentation also affect microbial fermentation. Studies have shown that in the biosynthesis of pinocembrin, high pH values favor upstream pathway catalysis, whereas low pH values favor downstream pathway catalysis. Thus, a two-stage pH control strategy has been proposed [19].

In one-step (2*S*)-pinocembrin production, expensive malonyl-CoA precursor malonate is needed, and morpholinopropane sulfonate is required to provide buffering capacity. To solve this problem, a CRISPR interference system has been established to effectively guide carbon flux to malonyl coenzyme A. In addition, by adjusting the pH value of the fermentation system, the yield of pinocembrin can be significantly improved [19]. In summary, the increase in pinocembrin production can be achieved by a stepwise metabolic engineering strategy in combination with malonyl-CoA engineering. In addition to the above strategies, a two-stage pH fermentation strategy and optimized strain culture should be combined to increase the production of pinocembrin. The synthesis of (2*S*)-pinocembrin can be achieved by assembling the Oc4CL1, OcCHS2, and MsCHI genes obtained in alfalfa into *E. coli* by a gene manufacturer [20].

## 3. Pharmacological Effects of Pinocembrin

**Figure 3:**
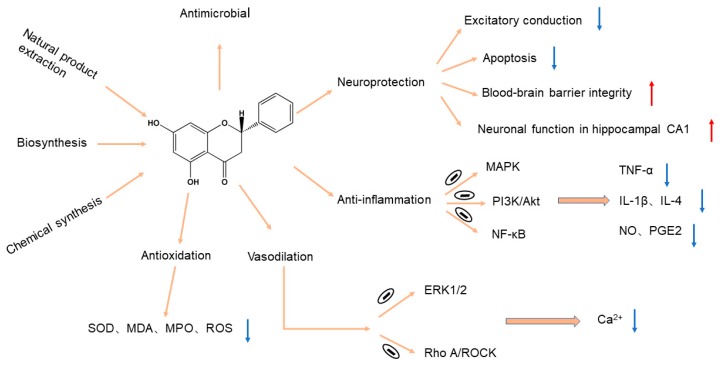
Pharmacological action and possible mechanisms of pinocembrin. Pinocembrin features a variety of pharmacological activities. This compound can inhibit the expression of pro-inflammatory factors by inhibiting mitogen-activated protein kinase (MAPK), phosphoinositide 3-kinase (PI3K)/AKT, and nuclear factor kappa B (NF-κB) signaling pathways, thereby exerting anti-inflammatory effects. Its vasodilation effect is achieved by inhibiting the ERK1/2 and Rho-associated protein kinase (ROCK) signaling pathways and then downregulating calcium ion concentration. The neuroprotective effects of pinocembrin mainly include the reduction in nerve excitability and neuronal apoptosis and enhances activity of cells in hippocampal CA1 region. Pinocembrin can down-regulate the contents of superoxide dismutase (SOD), malondialdehyde (MDA), myeloperoxidase (MPO), and ROS to achieve antioxidant effects.

Pinocembrin is mainly used for ischemic stroke treatment. However, recent studies have indicated that pinocembrin may exert therapeutic effects on Parkinson’s disease (PD) and Alzheimer’s disease (AD). Pinocembrin also exhibits anti-pulmonary fibrosis and vasodilating activities. Pinocembrin undergoes multiple mechanisms to perform its pharmacological effects. Furthermore, pinocembrin may alleviate BBB disruption and neurological injury via reducing the levels of reactive oxygen species (ROS) and inflammatory factors. In addition, pinocembrin may preserve mitochondrial integrity by activating the extracellular signal-regulated kinase/nuclear factor erythroid 2-related factor 2 (Erk1/2-Nrf2) pathway [21]. Pinocembrin also attenuates apoptosis by affecting the p53 pathway, thereby influencing the Bax-Bcl-2 ratio and the release of cytochrome C [22]. Antioxidant and anti-inflammatory activities serve as the basis for various pharmacological effects of pinocembrin (Figure 3). An in-depth understanding of its pharmacological activities and mechanisms of actions can aid in discovering new targets and potential therapeutic applications of pinocembrin.

### 3.1. Neuroprotective Activity

#### 3.1.1. Neuroprotective Effect in Cerebral Ischemia

Research shows that pinocembrin possesses the potential to become a drug for the treatment of ischemic stroke. Against the background trend of an increasingly aging population worldwide, the risk of cerebral ischemia is increasing, with 70% of survivors presenting physical disabilities [23]. Thus, developing drugs to treat cerebral ischemia is crucial. 

In vitro*,* pinocembrin can inhibit the reactivity of SN/L7 to 5-HT by reducing the excitatory conduction of synapses in co-cultured Aplysia SN/L7 neurons reversibly. This phenomenon is related to glutamate receptors in the postsynaptic membrane [24]. These events indicate that pinocembrin exerts certain effects on the nervous system. In an oxygen-glucose deprivation/reoxygenation model, pinocembrin can increase neuronal survival rates, decrease the amount of lactate dehydrogenase, and alleviate neurite length and apoptosis during reoxygenation [8]. These studies have initially shown that pinocembrin can alleviate nerve damage caused by cerebral ischemia in vitro. To verify whether pinocembrin also features neuroprotective effects in vivo, a rat model of focal cerebral ischemia has been applied. The result has shown that pinocembrin reduced the leakage of Evans Blue and sodium fluorescein, manifesting a protective action on BBB integrity [25]. Continued research has revealed that pinocembrin can improve the morphology of brain cortex, striatum, and hippocampal neurons in rats with acute focal cerebral ischemia/reperfusion. After ischemia reperfusion in rats, pinocembrin significantly suppressed the levels of neuronal specific enolase and S-100β protein in blood. In addition, the morphology of neurons in the hippocampal CA1 area improved, and the survival rate of neurons increased. All this evidence indicate that pinocembrin exhibits neuroprotective effects that prevent brain ischemia/reperfusion acute injury [5,26]. Moreover, the treatment time window of pinocembrin is wider than that of tissue plasminogen activator (t-PA). Pretreatment with pinocembrin shortly before t-PA infusion can significantly protect BBB function and improve neurological function after long-term ischemia, thereby improving the therapeutic effects of t-PA [27]. These data have shown that pinocembrin presents desirable neuroprotective effects and may be beneficial for the treatment of stroke in combination with t-PA.

What are the mechanisms of pinocembrin for neuroprotection? Studies have shown that the neuroprotective effects of pinocembrin include mitochondrial protection, inhibition of autophagy, anti-oxidation, anti-apoptosis, and other pharmacological effects. Pinocembrin reduces production of ROS, nitric oxide (NO), neuronal NO synthase (nNOS) and induces NO synthase (iNOS). Pinocembrin also increases the content of glutathione, thereby exerting an antioxidant effect and achieving neuroprotection [24]. The anti-inflammatory effect of pinocembrin also plays an important role in neuroprotection. Pinocembrin suppresses expression of inflammatory markers, such as tumor necrosis factor-alpha (TNF-α), interleukin-1 (IL)-beta, intercellular adhesion molecule-1, vascular cell adhesion molecule-1, iNOS, and aquaporin-4. In addition, pinocembrin inhibits the activation of microglials and astrocytes and downregulates the expression of matrix metalloproteinases (MMPs) in ischemic brain area. These events result in the protective action of pinocembrin on neurovascular units [28,29]. Anti-apoptosis and anti-autophagy of pinocembrin directly affect the total number of nerve cells. Treatment with pinocembrin can increase the viability of cells and attenuate apoptosis in a dose-dependent manner. Part of the mechanism of pinocembrin is to inhibit the release of p53, thereby affecting the Bax-Bcl-2 ratio and released amount of cytochrome C [20]. Pinocembrin decreases the expression of autophagy protein LC3 II and Beclin 1 and increases the level of p62, which are key proteins of autophagy in the hippocampus CA1 area. These results suggest that pinocembrin may achieve neuroprotection by inhibiting autophagy activity [30].

Pinocembrin can provide mitochondrial protection. Cerebral ischemia often causes mitochondrial damage in the brain. Thus, restoring mitochondrial function is important to fight this disease. Ca^2+^ overload consistently results in mitochondrial structure and function impairment. Pinocembrin can reduce the content of Ca^2+^ in mitochondria, thereby alleviating mitochondrial membrane swelling and reducing Mn-SOD activity. In addition, pinocembrin can reduce ATP synthesis and energy metabolism disorders caused by Ca^2+^ overload. Pinocembrin can also reduce ROS production in mitochondria and reduce electronic leakage of the NADH respiratory chain, improve the efficiency of oxidative phosphorylation, and promote mitochondrial respiratory function and synthesis of mitochondrial ATP [31,32,33]. The role of pinocembrin in mitochondrial protection depends in part on the activation of the Erk1/2-Nrf2 axis [20].

#### 3.1.2. Neuroprotective Effect of Pinocembrin in Alzheimer’s Disease and Parkinson’s Disease

AD is a central nervous system degenerative disease that seriously threatens the health of the elderly. Modern pharmacological studies have shown that common pathological features of AD include β-amyloid (Aβ) plaques, excessive phosphorylation of Tau protein to form neurofibrillary tangles, reduction of acetylcholine transmitters and inflammatory responses, and neuronal loss [34,35]. 

Pinocembrin has been found to affect cognitive function and protect nerve cells against Aβ-induced toxicity. Administration of pinocembrin in Aβ25–35-induced mice can preserve ultrastructural nerve fibers in the mouse brain and reduce neurodegeneration in the cerebral cortex, thereby improving cognitive function. The function of the nervous system is also affected by the interaction between Aβ and receptors of advanced glycation end products (RAGE). Pinocembrin significantly inhibits the upregulation of RAGE transcription and protein translation in vivo and in vitro and the inflammatory response following Aβ-RAGE interaction. These effects are achieved by inhibition of p38 MAPK–MAPK-activated protein kinase-2–heat shock protein 27 and stress-activated protein kinase/c-Jun N-terminal kinase–c-Jun pathway and NF-κB signaling pathway. Pinocembrin also improves the cholinergic system by conserving the ERK–cAMP-response element-binding protein–brain-derived neurotrophic factor pathway [36,37,38]. Treatment with pinocembrin reduced the damage induced by fibrillar Aβ (1–40) in human brain microvascular endothelial cells (hBMECs), and this finding may be related to inhibition of inflammation by pinocembrin. The related mechanisms may include the inhibited activation of MAPK, down-regulated the level of IκB kinase, decreased degradation of IκBα, and blocked nuclear translocation of NF-κB p65 by pinocembrin, thereby reducing the release of pro-inflammatory factors. Further studies have shown that pinocembrin can protect SH-SY5Y cells from Aβ (25–35)-induced neurotoxicity by activating the Nrf2/heme oxygenase-1 (HO-1) pathway [39]. The protective effect on mitochondria is also one of the anti-AD mechanisms of pinocembrin.

PD is characterized by the loss of dopaminergic neurons in the substantia nigra. Oxidative stress and mitochondrial dysfunction are possibly involved in the etiology of PD [40]. In a methyl-4-phenyl pyridinium (MPP^+^) -induced PD model, pinocembrin significantly reduced the loss of cell viability, production of intracellular ROS, apoptosis rate of cells, and activation of caspase-3 in SH-SY5Y cells. The related mechanisms may include the activation of ERK1/2 signaling pathways, enhancement of HO-1 expression, and suppression of MPP^+^-induced oxidative damage by pinocembrin. In addition, pinocembrin can alleviate MPP^+^-induced mitochondrial dysfunction by reducing mitochondrial membrane potential, down-regulating Bcl-2/Bax ratio, and inhibiting the release of cytochrome C [41,42]. In 6-hydroxydopamine (6-OHDA)-induced PD model, pinocembrin activates the expression of HO-1 and γ-glutamylcysteine synthetase via the Nrf2/antioxidant response element pathway in SH-SY5Y cells. As a result, the loss of cell viability and apoptosis rate induced by 6-OHDA in SH-SY5Y cells decreased, thereby reducing nerve damage [43].

### 3.2. Anti-Inflammation Activity

Pinocembrin significantly reduces the degree of cerebral edema and serum TNF-α and IL-1β levels in rats with focal cerebral ischemia reperfusion. The anti-inflammation effect may partly account for the mechanisms in ischemic stroke treatment [5]. In diabetic mice, pinocembrin protects neurons from inflammatory damage, thereby reducing their cognitive deficits [44]. Moreover, pinocembrin inhibits the inflammation of allergic airways induced by ovalbumin (OVA) in mice and significantly reduces the content of Th2 cytokines, IL-4, IL-5, and IL-13 in broncho-alveolar lavage fluid and OVA-specific antibody IgE in serum. A possible mechanism involves the inhibition of IκBα and NF-κB p65 phosphorylation. These findings indicate that pinocembrin features the potential to become a natural antiallergic drug [45]. The degradation of extracellular matrix induced by MMPs is an important cause of cartilage destruction. Pinocembrin inhibits the expression of MMP-1, MMP-3, and MMP-13 at both mRNA and protein levels in human chondrocytes [46]. In lipopolysaccharide-induced inflammation, pinocembrin inhibits the production of TNF-α, IL-1β, NO, and PGE2 by suppressing PI3K/Akt/NF-κB signaling pathway [47,48]. The mechanism by which pinocembrin inhibits inflammation includes inhibition of the MAPK and NF-κB signaling pathway. Through the above routes, pinocembrin can reduce the release of pro-inflammatory cytokines [37]. 

### 3.3. Antioxidation Activity

The anti-oxidation effect of pinocembrin is the basis for treatment of numerous diseases using this compound. The antioxidation activity of pinocembrin includes neuroprotection and mitochondrial protection against cerebral ischemia, AD, PD, and other diseases. In the global cerebral ischemia model, pinocembrin can reduce brain tissue damage by reducing the compensatory activity of SOD and reducing MDA levels and MPO activity in a dose-dependent manner [49]. In oxidative stress injury evoked by CCl_4_, pinocembrin restores liver transaminases and total cholesterol to normal levels through the inhibition of reduced glutathione depletion and lipid peroxidation and elevation of superoxide dismutase (SOD) [6]. Pinocembrin can also protect human aortic endothelial cells from ox-low-density lipoprotein (LDL)-induced injury. The mechanisms may relate to ROS reduction induced by ox-LDL [50]. The nephrotoxicity induced by gentamicin can be alleviated by pinocembrin due in part to its antioxidant effect [51]. These findings have indicated that pinocembrin reduces the degree of atherosclerosis and is a promising antioxidant.

### 3.4. Antimicrobial Activity

Pinocembrin possesses antibacterial, antifungal, and antiparasitic effects. A study has shown that pinocembrin features anti-*Staphylococcus aureus* action both in vitro and in vivo [52]. Pinocembrin presents significant inhibition of zoospore mobility and mildew development and thus could be used as a natural antifungal product [53]. Pinocembrin significantly suppressed parasitemia in *Plasmodium berghei*-infected mice [54]. More importantly, pinocembrin plays a highly reversible role as antimicrobial. Substituent groups will affect the pharmacological activities of pinocembrin. Introducing oleyl or linoleoyl in the seventh carbon, the derivatives showed high inhibitory effects on bacterial proliferation, with minimum inhibitory concentration values of 32 µg/mL against *Staphylococcus aureus* [55].

### 3.5. Vasodilation Activity

Pinocembrin has been observed to inhibit angiotensin II (Ang II)-induced vasoconstriction in aortic rings of rats. In the docking model, pinocembrin binds effectively to the active site of Angiotensin II receptor type 1(AT1R), thereby inhibiting Ang II induced Ca^2+^ release and Ca^2+^ influx. These inhibitory effects may be related to the reduction of Ang II-induced increase in Ca^2+^ and ERK1/2 activation via blocking of AT1R [56]. Pinocembrin can induce endothelium-independent relaxation in rat aortic rings, and the mechanism is at least partly due to the blockade of the Rho A/ROCK pathway [57,58].

### 3.6. Hepatoprotection Activity

In treatment of liver fibrosis, inactivating hepatic stellate cells (HSCs) has been an effective therapeutic strategy. It has been discovered that pinocembrin inhibits the expressions of fibrotic markers in LX-2 cells and rat HSCs (HSC-T6). Pinocembrin can reduce ROS accumulation by elevating the expression and activity of silent mating type information regulation 2 homolog 3 (SIRT3) and then activating SOD2. In addition, pinocembrin inhibits the PI3K/Akt signaling pathway, resulting in decreased production of transforming growth factor-beta and inhibition of transcriptional factors Sma- and Mad-related protein (Smad) nuclear translocation. Moreover, pinocembrin activates glycogen synthase kinase 3β by acting on SIRT3, thereby enhancing the degradation of Smad protein [10]. Pinocembrin-7-*O*-[3ʺ-*O*-galloyl-4ʺ,6ʺ-hexahydroxydiphenoyl]-β-glucose is the derivative of pinocembrin, and it significantly contributes to the hepatoprotective effects of the latter [59]. This finding indicates that pinocembrin can be used as a drug candidate for the treatment of liver diseases. 

### 3.7. Others

Recent studies have also reported that pinocembrin features an anti-tumor effect. Pinocembrin up-regulates the levels of caspase 3 and LC3-II in MDA-MB-231 cells, induces apoptosis and autophagy, and exhibits potential anticancer effect [60]. In melanoma, pinocembrin can induce endoplasmic reticulum (ER) stress-mediated apoptosis and suppress autophagy, showing its potentiality for melanoma treatment. Pinocembrin induces ER stress via the inositol-requiring endonuclease 1 α/X-box binding protein 1 pathway and then triggers caspase-12/-4 mediated apoptosis by suppressing autophagy through the activation of PI3K/Akt/mTOR pathway [9]. Pinocembrin also reduced ventricular arrhythmias in I/R rats by enhancing Na^+^-K^+^ ATPase and Ca^2+^-Mg^2+^ ATPase activity and up-regulating Cx43 and Kir2.1 protein expression [61].

Furthermore, studies have shown that pinocembrin can prevent kidney damage caused by diabetes, but when the kidney is damaged, pinocembrin will aggravate the organ’s condition [62]. This finding indicates that pinocembrin can be used as a preventive agent for kidney damage before injury. Pinocembrin may enhance the activities of hexokinase and pyruvate kinase via the Akt/mTOR signaling pathway, thereby improving insulin resistance [63]. In daily life, given its antibacterial and antioxidant activities, pinocembrin can be used as mouth cleaning agent and sunscreen [7,64]. 

## 4. Pharmacokinetic Profiles of Pinocembrin

### 4.1. Transport Features Across Blood–Brain Barrier (BBB) In Vitro

Cultured rat BMECs have been used as an in vitro BBB model. The findings have shown the uptake of pinocembrin in a time- and concentration-dependent manner. Passive transport may be the main process for pinocembrin to pass through the BBB, whereas P-gp is likely to cause little effect on the transport process of pinocembrin. Furthermore, pinocembrin may show no effect on the functional activity and protein expression of the P-gp transporter at the BBB [11].

### 4.2. Pharmacokinetic (PK) Profiles In Vivo

#### 4.2.1. PK Profiles in Rats

In rats, pinocembrin exhibits a large volume of distribution (V_d_) and a short half-life (T_1/2_) and is easily metabolized in the body (Table 1). After intravenous injection of pinocembrin in rats, the cumulative excretion scores of drug prototypes excreted from urine and excrement in 72 h totaled 6.99% ± 5.97% and 59.17% ± 22.13%, respectively. In rats, the drug is metabolized rapidly, and 40.6% ± 23.52% of the metabolites are excreted by urine [65]. In racemic delivery (±) of pinocembrin to rats (20 mg/kg, intravenously (iv.)), the concentration–time profile of pinocembrin followed a biexponential pattern, which indicates differences in the in vivo process of different configurations of pinocembrin. *S*-Pinocembrin and *R*-pinocembrin could be detected in serum. Similar values of V_d_ are observed between enantiomers, and both enantiomers exhibit a serum half-life (T_1/2_) of about 15 min in rats. After oral administration, pinocembrin is rapidly glucuronidated. The peak concentration of S-pinocembrin glucuronide and *R*-pinocembrin glucuronide measure 140 and 160 µg/mL, respectively. The main metabolic mode of pinocembrin is phase II metabolism. The large V_d_ coupled with the short serum T_1/2_ suggests the extensive distribution of pinocembrin into the tissues [66,67,68].

#### 4.2.2. PK Profiles in Humans

Pinocembrin is well absorbed and widely distributed in the human body. In a single-dose study, five dose groups have been established, and the mean peak plasma pinocembrin concentration has been obtained at the end of 30 min infusion. T_1/2_ is similar in the five dose groups and ranges from 40 min to 55 min. At 4 h after administration, the cumulative excretion rate of pinocembrin in the urine reaches a plateau, and the level of urine and fecal excretion of pinocembrin is extremely low, with each dose group yielding similar values. The data show that pinocembrin is easily metabolized in vivo. The compound is mainly metabolized into two metabolites in the human body, sulfonic products and glucuronide products, among which 5-hydroxy-flavanone-7-*O*-sulfonate and 5-hydroxy-flavanone-7-*O*-β-D glucuronic acid can be synthesized by artificial synthesis. The V_d_ of pinocembrin approximates 136.6 ± 52.8 L, and the clearance (CL) rate equals 2.0 ± 0.31 L/min, indicating that pinocembrin is well absorbed and widely distributed in human body. The PK features of pinocembrin under multiple dose are similar to those observed in single-dose studies, showing no evidence of accumulation. Pinocembrin is well tolerated when administered iv. to healthy adults [69,70,71,72]. Although the absorption/elimination process of pinocembrin occurs rapidly, and no serious accumulation exists in the body, the related drug interaction still deserves attention. In a study, 6β-hydroxylation of testosterone was used as a labeling reaction for CYP3A4 activity. The resulting product was determined by high-performance liquid chromatography in conjunction with diode array detector. Metabolism, time dependence, and direct inhibition were tested to determine if inhibition of CYP3A4 activity is reversible or irreversible. The result showed that pinocembrin irreversibly inhibited the metabolic activity of the CYP3A4 enzyme, decreasing the enzyme activity by 50% [73]. In addition, pinocembrin has been shown to inhibit hOATP2B1 and hOATP1A2, with IC_50_ of 37.3 ± 1.3 and 2.0 ± 1.7 µM, respectively, affecting the intake of statins [74]. In addition, racemic pinocembrin reveals the inhibitory activity of CYP2D6 at low concentrations. At 0.01 and 0.1 µM, the inhibition of CYP2D6 approximates 50% compared with the positive control [67]. Given the inhibitory effect of pinocembrin on drug-metabolizing enzymes, drug interactions should be considered when using the compound.

## 5. Conclusions and Prospects

Although pinocembrin is widely found in honey and various plants, the yield of natural extraction remains insufficient. Biosynthesis fills the gap in this area. In this review, we summarized the progress in biosynthesis of pinocembrin, relying on genetic engineering technology to construct engineered bacteria to achieve the synthesis of pinocembrin from glucose. The production of pinocembrin can be considerably improved by regulating the metabolism of engineered bacteria and regulating pH and energy supply of the fermentation system. 

Pinocembrin exerts certain effects on ischemic stroke, PD, AD, solid tumors, and some other diseases. In central nervous system diseases, pinocembrin can reduce the release of inflammatory factors by inhibiting multiple signaling pathways, such as MAPK and PI3K/AKT. Pinocembrin can also reduce the release of NO, ROS, nNOS, and iNOS, thus playing an antioxidant role. The activation of ERK1/2-Nrf2 axis is one mechanism of mitochondrial protection by pinocembrin. In addition, this compound can increase the number of nerve cells by inhibiting their autophagy. Given its wide range of pharmacological activities, pinocembrin can be linked to disease production mechanisms, such as network pharmacology and molecular target docking, to explore its application in some other diseases.

Pinocembrin may easily cross the BBB due to its low molecular weight and good liposolubility. Several studies have shown that pinocembrin features an anti-tumor effect, undergoing passive transport when passing the BBB; this property can be used for the treatment of drug-resistant brain tumors. However, the anti-tumor mechanism of pinocembrin remains unclear, thus requiring further research. Numerous diseases are often associated with inflammation and oxidative damage during production and development. Pinocembrin performs significant anti-oxidant and anti-inflammatory activities, indicating that it possesses the potential to treat a variety of diseases and thus needs further research. In addition, pinocembrin exhibits an inhibitory effect on various drug-metabolizing enzymes and transporters. Thus, drug interactions should be considered when using this compound to ensure drug safety. Pinocembrin is in a phase II clinical trial and requires more in-depth studies.

## Figures and Tables

**Table 1:** Summarizes the PK parameters of pinocembrin.

Subject	Mode of Administration	Dose (mg/kg)	AUC (h*µg/mL)	V_d_ (L/kg)	CL_total_ (L/h/kg)	T_1/2_ Serum (h)	References
SD rats	iv.	10	*S*-1.821 ± 0.211; *R*-1.876 ± 0.427	*S*-1.758 ± 1.313; *R*-1.793 ± 0.805	*S*-5.527 ± 0.641; *R*-5.535 ± 1.217	*S*-0.212 ± 0.140; *R*-0.223 ± 0.083	[67]
SD rats	iv.	10	*S*-1.83 ± 0.092; *R*-1.876 ± 0.312	*S*-1.46 ± 0.591; *R*-1.80 ± 0.271	*S*-5.44 ± 0.287; *R*-5.83 ± 0.865	*S*-0.262 ± 0.071; *R*-0.263 ± 0.027	[66]
SD rats	po.	100	*S*-570 ± 21.7; *R*-531 ± 82.1	*S*-3.80 ± 1.34; *R*-5.14 ± 1.81	*S*-2.82 ± 0.084; *R*-2.83 ± 0.844	*S*-20.3 ± 8.41; *R*-27.1 ± 18.8	[66]
SD rats	iv.	10	0.686.1 ± 0.0651	48.7 ± 19.6	15.5 ± 1.4	2.14 ± 0.68	[68]
SD rats	po.	50	0.518 ± 0.170	478 ± 213	110 ± 31.4	3.11 ± 1.21	[68]
Human	iv.	20 (mg)	10.3381 ± 1.5394 (min μg/mL)	136.6 ± 52.8 (L)	2.0 ± 0.3 (L/min)	0.79 ± 0.23	[71]

AUC: area under the curve; CL: clearance; iv.: intravenous; T_1/2_: half-life; V_d_: volume of distribution.
